# Supplementary material for: Uterine Septum with or without Hysteroscopic Metroplasty: Impact on Fertility and Obstetrical Outcomes—A Systematic Review and Meta-Analysis of Observational Research
Source: J Clin Med. 2022 Jun 8;11(12):3290. doi: 10.3390/jcm11123290 (PMC9224595; doi:10.3390/jcm11123290)
Supplement: Supplementary file 1 [file jcm-11-03290-s001.zip › jcm-1731504-supplementary.pdf]

LEGEND OF TABLES

Supplementary Table S1. Complications of surgical procedures in "Treated uterine septum versus controls (untreated septum)" section.

**Study**	**Sample Size: case/control**	**Complications**
**Heinonen et al. 1997, J Am Assoc Gynecol Laparosc**	19/19	Three patients:<br>- One uterine perforation but surgical intervention was unnecessary.<br>- Two patients with abnormal bleeding treated by inserting a Foley catheter into the uterine cavity.
**Valli et al. 2004, J Am Assoc Gynecol Laparosc**	28/15	One case of a small fundal perforation that required a laparoscopic suture
**Ban-Frangez et al. 2009, Eur J Obstet Gynecol**	106/212	N/A
**Lin et al. 2009, Int J Gynaecol Obstet**	21/15	No complications
**Pang et al. 2011, Int J Gynecol Obstet**	46/32	N/A
**Tonguc et al. 2011, Int J Gynecol Obstet**	102/25	N/A
**Sugiura-Ogasawara et al. 2014, Journal of Obstetrics and Gynaecology**	109/15	N/A
**Rikken et al. 2020, Human Reproduction**	151/106	Seven patients:<br>- Three women with uterine perforation, in<br>- One woman with intravasation<br>- Three women with excessive bleeding
**Whelan et al. 2020, American College of Obstetricians and Gynecologists**	21/11	N/A

Supplementary Table S2. Complications of surgical procedures in "Before and after septum removal" section.

Sudy	Simple size	Complications
**Valle et al. 1986, Obstet Gynecol**	124	No complications
**Guarino et al. 1989, Acta Eur Fertil**	19	N/A
**Choe et al. 1992, Fertil Steril**	14	Two patients:<br>- One case was complicated by a small perforation that did not require any treatment of the uterus during procedure.<br>- Second case developed uterine synechiae in the left cornual area postoperatively on HSG.
**Grimbizis et al. 1998, Hum Reprod**	57	During the procedure, two operations were complicated by small perforations that did not require any further treatment.
**Jourdain et al. 1998, Int J Gynaecol Obstet**	17	No complications
**Porcu et al. 2000, Eur J Obstet Gynecol Reprod Biol**	63	One case of abnormal bleeding was controlled by endoscopical coagulation.
**Venturoli et al. 2002, Arch Gynecol Obstet**	141	No complications
**Saygili-Yilmaz et al. 2003, Arch Gynecol Obstet**	361	Fifteen patients:<br>-Five patients with fluid overload syndrome<br>-Ten patients with uterine perforation (five of these cases were repaired by laparoscopy right after the septum incision. The other five however, had to be repaired by laparotomy due to the size of the perforation >1 cm).
**Yang et al. 2006, Photomed Laser Surg**	46	N/A
**Wang et al. 2009, Fertil Steril**	25	No complications
**Roy et al. 2011, Arch Gynecol Obstet**	152	Five patients.<br>- One patient with difficult dilatation leading to cervical injury<br>- Two patients with uterine perforation (laparoscopy by bipolar coagulation).<br>- Two patients had excessive bleeding at the end of the procedure which was managed by the tamponade of Foley's
**Sendag et al. 2010, Clin Exp Obstet Gynecol**	30	N/A
**Gergolet et al. 2012, Reproductive BioMedicine Online**	72	No complications
**Bendifallah et al. 2013, J Minim Invasive Gynecol**	128	Four patients with uterine perforation
**Gundabattula et al. 2014, J obstet Gynecol**	124	N/A

**Paradisi et al. 2014, Arch Gynecol Obstet**	112	No complications
**John et al. 2015, J Minim Invasive Gynecol**	286	One patient with uterine perforation
**Freud et al. 2015, J Matern Fetal Neonatal Med**	28	N/A
**Wang et al. 2019, Medicine (Baltimore)**	121	No complications

LEGEND OF FIGURES:

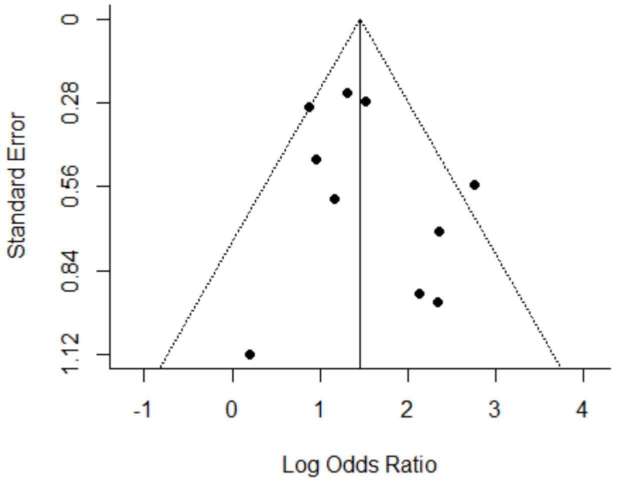

Supplementary Figure S1. Funnel plot of studies comparing spontaneous abortions in I-II trimesters between septate uterus vs. controls (no septate uterus).

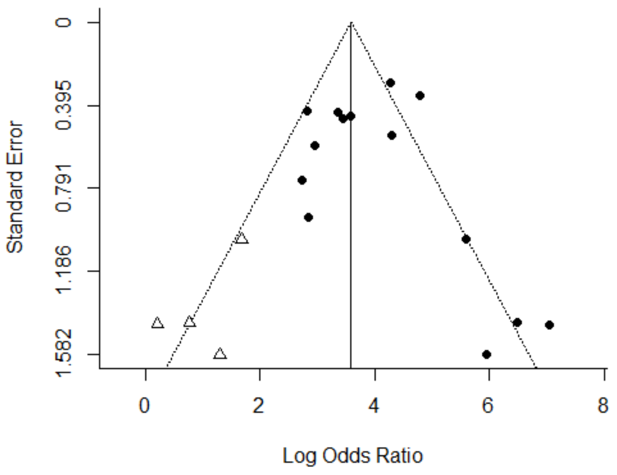

Supplementary Figure S2. Funnel plot of studies comparing live birth rate before vs. after the removal of septate uterus

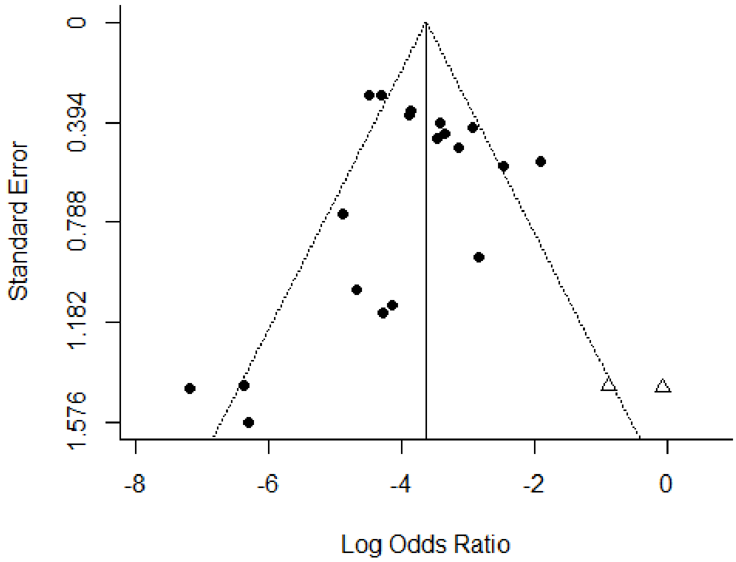

Supplementary Figure S3. Funnel plot of studies comparing spontaneous abortions in I-II trimesters before vs. after the removal of septate uterus.

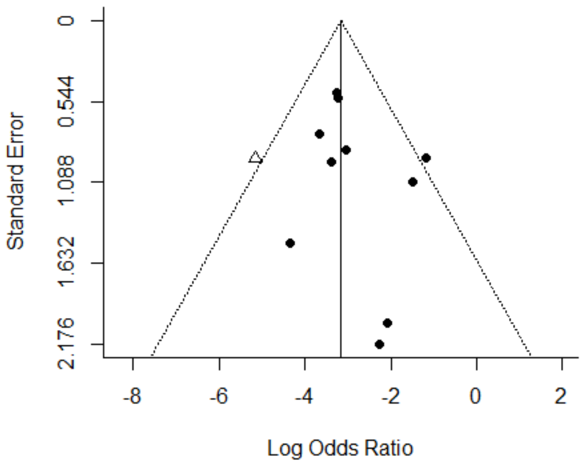

Supplementary Figure S4. Funnel plot of studies comparing preterm labour before vs. after the removal of septate uterus
